# A manually curated database of tetrapod mitochondrially encoded tRNA sequences and secondary structures

**DOI:** 10.1186/1471-2105-8-441

**Published:** 2007-11-14

**Authors:** Konstantin Yu Popadin, Leila A Mamirova, Fyodor A Kondrashov

**Affiliations:** 1 Institute for Information Transmission Problems RAS, Bolshoi Karetny pereulok 19, Moscow 127994, Russia; 2 Section on Ecology, Behavior and Evolution, Division of Biological Sciences, University of California at San Diego, 2218 Muir Biology Building, La Jolla, CA 92093, USA

## Abstract

**Background:**

Mitochondrial tRNAs have been the subject of study for structural biologists interested in their secondary structure characteristics, evolutionary biologists have researched patterns of compensatory and structural evolution and medical studies have been directed towards understanding the basis of human disease. However, an up to date, manually curated database of mitochondrially encoded tRNAs from higher animals is currently not available.

**Description:**

We obtained the complete mitochondrial sequence for 277 tetrapod species from GenBank and re-annotated all of the tRNAs based on a multiple alignment of each tRNA gene and secondary structure prediction made independently for each tRNA. The mitochondrial (mt) tRNA sequences and the secondary structure based multiple alignments are freely available as Supplemental Information online.

**Conclusion:**

We compiled a manually curated database of mitochondrially encoded tRNAs from tetrapods with completely sequenced genomes. In the course of our work, we reannotated more than 10% of all tetrapod mt-tRNAs and subsequently predicted the secondary structures of 6060 mitochondrial tRNAs. This carefully constructed database can be utilized to enhance our knowledge in several different fields including the evolution of mt-tRNA secondary structure and prediction of pathogenic mt-tRNA mutations. In addition, researchers reporting novel mitochondrial genome sequences should check their tRNA gene annotations against our database to ensure a higher level of fidelity of their annotation.

## Background

Mitochondrially encoded tRNAs (mt-tRNAs) are an excellent object of study for researchers in several fields for a variety of reasons. The primary reason is the wide variety of available completely sequenced mitochondrial genomes, which provides a large data sample from a broad phylogenetic background. Besides the obvious availability factor, mt-tRNAs show several unusual properties. mt-tRNAs are of particular interest to structural biologists, since the secondary structure of the mt-tRNAs is not as conserved as that of their nuclear encoded counterparts [1], and some mt-tRNAs in several lineages show accelerated rates of secondary structure evolution [2]. Although some changes of the secondary structure may be of limited use as a phylogenetic marker the observation of parallel loss of the D-loop structure [1-3] may lead to our understanding of the broader issues associated with parallel evolution of secondary structure.

The evolution of secondary structure in mt-tRNAs is also coupled with rapid compensatory evolution that is aimed at the conservation of the secondary structure stability [4]. Indeed, as much as 50% of the substitutions in mt-tRNAs may be compensatory [4], and further study of these molecules may shed light on the selective pressure governing compensatory evolution. Compensatory changes have also been observed on a larger scale, with the import of nuclear coded Lys-tRNA was shown to compensate for complete loss of mt-Lys-tRNA in metatherians [5]. Also, mt-tRNAs appear to be targets for post-transcriptional RNA modification mechanisms [6] including instances of RNA editing where modification of nucleotides that would otherwise be damaging to function or structure takes place [7].

Although mt-tRNAs span only 10% of the entire mitochondrial genome, they appear to be "hotspots" of disease-causing mutations, such that 50% of all pathogenic mutations that have described for the mitochondria have been localized to one of the mt-tRNAs [8]. In addition, some mt-tRNAs appear to harbor more disease-causing variants than others [8,9]. While no explanation for these observations has been adequately tested, they underline the medical importance of mt-tRNAs [10,11]. The availability of secondary structure information [12,13] and evolutionary information including compensatory changes [13,14] have made progress in the identification and possible treatment of deleterious variants in mt-tRNAs [14,15]. Thus, a compilation of a manually curated database of mt-tRNAs incorporating a multiple alignment of genes from many closely related species and an independent secondary structure prediction, would serve to advance structural, evolutionary and medically relevant studies of mt-tRNAs and aid in the annotation of mt-tRNAs in newly sequenced mitochondrial genomes.

## Construction and content

We obtained complete tetrapod mitochondrial genomes from GenBank [16] using the Entrez search system [17] with "tetrapoda AND complete AND genome" in as the key entry and setting the Limits option of the Entrez search to mitochondrial sequences. A total of 277 different tetrapod genomes were obtained, including 148 mammalian, 53 amphibian and 76 saurosopoda genomes (all GenBank files used are available at the database website). We then obtained the sequence of all annotated tRNAs and their flanking regions using a Perl script. All of the mt-tRNA sequences were aligned with the muscle program [18] and then manually corrected, using the previously available alignment based on secondary structure information from Helm *et al*. [1] and two more extensive datasets based on secondary structure alignments [4,14]. Due to extensive divergence between tetrapods we made no attempt to align sequences in D- and T- loops.

Obvious errors in the sequence and the alignment were changed manually. Among large-scale errors annotation of the tRNA on the wrong strand was the most common (71 cases out of 6060 tRNAs) followed by labeling of the tRNA as transporting the wrong amino acid or complete omissions (20 cases out of 6060 tRNAs). However, most errors were more subtle, such as an omission or addition of several nucleotides in the flanks of the gene (690 cases out of 6060 tRNAs), that were corrected only through the analysis of secondary structure information coupled with the availability of a multiple alignment for the entire gene.

Since mt-tRNAs are not as conserved in sequence and structure as nuclear tRNAs [1-3] a compilation of a multiple alignment alone is insufficient for an accurate secondary structure annotation. Thus, we have chosen to add a secondary structure prediction using mfold [19] in addition to that produced by the multiple alignment. We predicted secondary structure using mfold web server and a Perl script to automate the Web routines. We ran mfold specifying pairing constraints for stem structures predicted from the earlier step of the multiple alignment. We made no attempt to restrict pairing of sequence sections predicted to be loops. If the above constraints (binding of some nucleotide pairs) returned implausible tRNA structures or no structures at all, the alignment and secondary structure were modified and mfold was ran again to test the plausibility of the new structure. Finally we ranked each type of tRNA molecules according to their free energies and checked by hand 25% of tRNAs with the highest free energy. Since we have not constrained loop sizes during these iterations some loop sizes decreased and stem sizes increased, leading to increased stability of tRNA molecules.

Empirically we observed the following constraints of tRNA secondary structure folding as performed by mfold: 1) minimum loop size was never less than 3 nucleotides and 2) WC and GU pairs were not formed if both of the neighbor nucleotides did not participate in pairing. A few mt-tRNA mfold secondary structures predictions that did not conform to the expected cloverleaf were manually rechecked and the alignment altered until the mfold prediction yielded a cloverleaf-like structure. In addition, some mt-tRNAs, including the mt-tRNA^SerAGY ^in all species [1] and several reptilian and nine banded armadillo mt-tRNAs^Cys ^[1-3] showed secondary structures that differed from the expected cloverleaf structure due to the loss of the D-stem structure, which is a structural evolutionary change particularly common in mt-tRNAs [2].

## Utility and Discussion

Most tetrapod mitochondrial genomes code for 22 different tRNAs with the exception of Metatherians that have lost the mt-tRNA^Lys ^[5]. In addition, some tetrapod mitochondrial genomes that were labeled as complete were only partially finished, such that seven mammalian genomes did not have sequences for tRNA^Phe ^(*Dromiciops gliroides*, *Metachirus nudicaudatus*, *Macrotis lagotis*, *Notoryctes typhlops*, *Perameles gunnii*, *Pseudocheirus peregrinus *and *Thylamys elegans*) and five mammals did not have the sequence for tRNA^Pro ^(*Arctocephalus forsteri*, *Dromiciops gliroides*, *Macrotis lagotis*, *Perameles gunnii *and *Thylamys elegans*). Thus, our database contains complete manually curated sequence and secondary structure information for 6060 mitochondrially encoded tRNA molecules.

**Figure 1:**
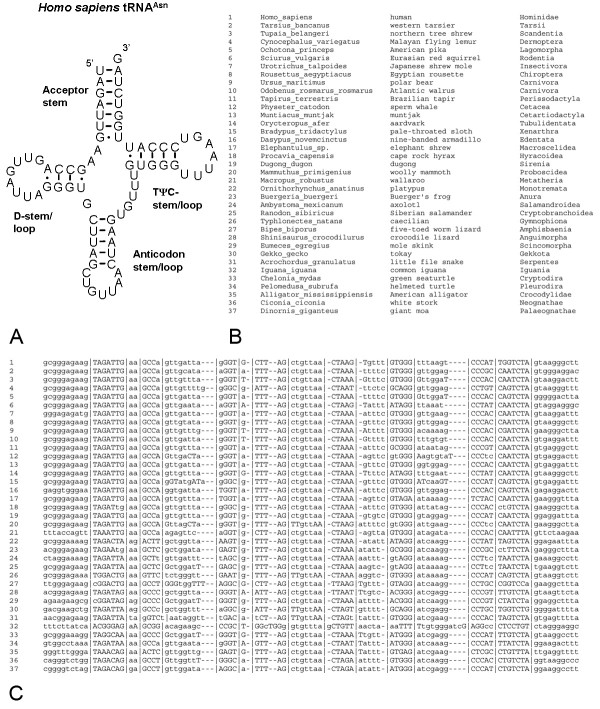
The secondary structure of the human mt-tRNA^Asn ^(a) and the multiple alignment with annotated secondary structure for selected species of mt-tRNA^Asn ^(b, c). The "|" characters separate the loops and stems based on the accepted basic secondary structure of mt-tRNAs form Helm *et al*. (2000) while capital letters denote those nucleotides that are predicted by mfold to participate in WC or GU pairing in stem structures.

**Figure 2:**
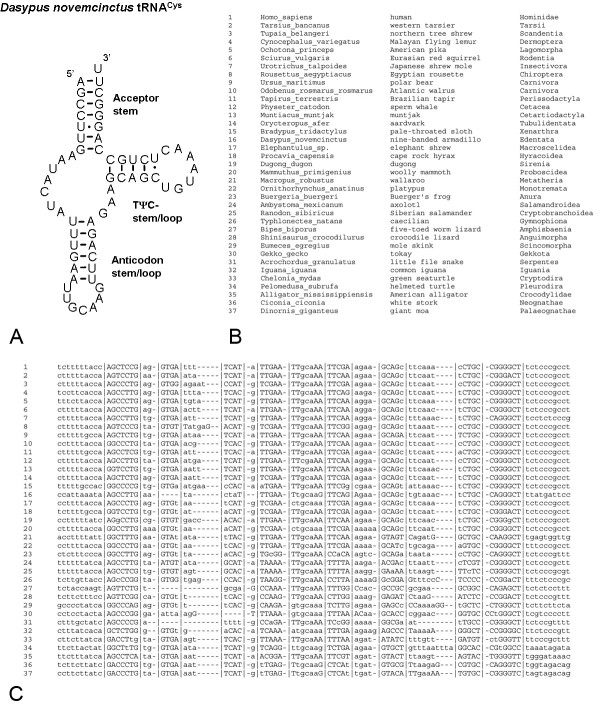
The secondary structure of the the nine-banded armadillo mt-tRNA^Cys ^(a), and the multiple alignment with annotated secondary structure for selected species of mt-tRNA^Cys ^(b, c). The "|" characters separate the loops and stems based on the accepted basic secondary structure of mt-tRNAs form Helm *et al*. (2000) while capital letters denote those nucleotides that are predicted by mfold to participate in WC or GU pairing in stem structures. The secondary structure of mt-tRNA^SerAGY ^in our database resembles the one of the nine-banded armadillo mt-tRNA^Cys ^(c).

Our database is available in 22 text files, one for each tRNA, with sequences of the 277 different species presented in the same order in each file. The order of the species in the alignment is the same for each mt-tRNA gene and roughly recapitulates the tetrapod phylogeny. Each file in the database includes the species common and scientific names, basic phylogenetic information and a multiple alignment of the tRNA with unaligned flanking sequence and annotated secondary structure (Figure 1 and 2). The "|" characters in the alignment delineate the conserved secondary structure prediction that was made using the alignment of all tRNA genes. The capital and lowercase letters in the files represent paired nucleotides according to the secondary structure prediction that was made with mfold. The two methods of secondary structure prediction generally showed similar results but small differences were common. For example, according to the mfold prediction many species in the tRNA^Asn ^gene form 3 WC pairs in the D-stem, while the classical tRNA structure supported by the alignment predicts 4 interacting nucleotides in this stem (Figure 1b). The value of showing separate predictions made by the alignment and the secondary structure is more evident in complicated cases, such as the case of the anticodon stem in the tRNA^Asn ^of the common iguana. In this case the alignment delineates the overall area where the anticodon stem should be formed, while mfold predicts which nucleotides form WC pairs in the structure (Figure 1b). Our database has a simple tab-delimited format with a set number of species in exactly the same order in each file making it especially useful for those researchers that wish to use our database in batch by parsing information on the secondary structure from our files.

The first database of mammalian mt-tRNAs which we used as a kernel in our alignment reports only mammalian species, it does not report any secondary structure that is independent of a multiple alignment and excludes complicated cases, such as the loss of D-stems [1]. Another, more current database that includes nuclear and mitochondrial tRNAs from the entire diversity of life forms has been, unfortunately, derived automatically [20] and is unlikely to be useful to researchers requiring a high level of sequence and structure annotation fidelity. In addition, both of these databases are difficult to use in batch mode, as they do not represent their results in a parsing-friendly format. Thus, our database is likely to be more useful for researchers that require a low level of annotation error, a phylogenetically diverse sample or prefer to work with many tRNA genes in simple text files. However, our database is not tailored to the needs of researchers that require a graphical interface for their work.

In the course of re-annotation and the compilation of a secondary-structure based multiple alignment, we have modified the annotation of the mt-tRNA gene location for 13% of all mt-tRNAs presented in our database. Such a high error rate in the annotation of such seemingly simple molecules as mt-tRNAs underscores the importance of availability of manually annotated databases such as the one reported here. In particular, we suggest for researchers reporting novel mitochondrial genome sequences to check their tRNA gene annotations against our database to ensure a higher level of fidelity of their annotation. Manually curated databases have an inherent advantage of a lower error rate than automatically created ones. However, a manual assembly of such an extensive database as the one reported here is a resource-intensive enterprise, and it is unlikely that the current database will be considerably expanded using the same manual approach. Rather the aim for the further development of this resource is to use the alignments reported here as a basis for further automatic enlargement.

## Conclusion

We report a secondary structure based multiple alignment of 6060 mt-tRNAs from 277 tetrapod species. In the course of our work, we have re-annotated a large fraction of mt-tRNA genes, and manually checked all secondary structure predictions. We expect that our database will facilitate further research of mitochondrially encoded tRNAs from a structural, evolutionary and medical perspectives. Currently, mammalian mitochondrial tRNAs are thought to have a high level of similarity to the canonical tRNA secondary structure [1]. However, an analysis of exceptions to the canonical tRNA structures among the vertebrate mt-tRNAs, which is made possible with the database reported here, has not been undertaken. The evolutionary implications of compensations on a molecular level have been investigated previously [4], however, the study of CPDs in mt-tRNAs has been performed only on mammalian mt-tRNAs. Finally, prediction of pathogenic mutations in mt-tRNAs relies heavily on evolutionary conservation [13,14] and the availability of a secondary structure-based alignment of an expanded set of species may contribute to a more accurate prediction of the phenotypic consequences of mt-tRNA mutations.

## Availability and requirements

Project name: mt tRNA tetrapod database;

Project home page: ;

Operating system(s): Platform independent;

Programming language: none

License: no restriction;

Any restrictions to use by non-academics: no restriction.

## Authors' contributions

KYuP, LAM and FAK conceived the construction of the database, and participated in the construction of the initial and final alignments, corrected erroneously annotated tRNAs and were involved in secondary structure prediction. FAK drafted the paper, and all authors read and approved the final manuscript.
